# mixtur: An R package for designing, analysing, and modelling continuous report visual short-term memory studies

**DOI:** 10.3758/s13428-021-01688-1

**Published:** 2022-01-31

**Authors:** James A. Grange, Stuart B. Moore

**Affiliations:** School of Psychology, Dorothy Hodgkin Building, Keele University, Keele, ST5 5BG UK

**Keywords:** Visual short-term memory, Mixture modelling, R, Simulation

## Abstract

Visual short-term memory (vSTM) is often measured via continuous-report tasks whereby participants are presented with stimuli that vary along a continuous dimension (e.g., colour) with the goal of memorising the stimulus features. At test, participants are probed to recall the feature value of one of the memoranda in a continuous manner (e.g., by clicking on a colour wheel). The angular deviation between the participant response and the true feature value provides an estimate of recall precision. Two prominent models of performance on such tasks are the two- and three-component mixture models (Bays et al., *Journal of Vision*, *9*(10), Article 7, 2009; Zhang and Luck, *Nature*, *453*(7192), 233–235, 2008). Both models decompose participant responses into probabilistic mixtures of: (1) responses to the true target value based on a noisy memory representation; (2) random guessing when memory fails. In addition, the three-component model proposes (3) responses to a non-target feature value (i.e., binding errors). Here we report the development of mixtur, an open-source package written for the statistical programming language R that facilitates the fitting of the two- and three-component mixture models to continuous report data. We also conduct simulations to develop recommendations for researchers on trial numbers, set sizes, and memoranda similarity, as well as parameter recovery and model recovery. In the Discussion, we discuss how mixtur can be used to fit the slots and the slots-plus-averaging models, as well as how mixtur can be extended to fit explanatory models of visual short-term memory. It is our hope that mixtur will lower the barrier of entry for utilising mixture modelling.

Visual short-term memory (vSTM) refers to the system involved with the storage of visual information over brief periods of time (Phillips, 1974). While the duration of iconic memory lasts on the order of milliseconds (Rensink, 2014; Sperling, 1960), vSTM stores information for considerably longer periods of time (on the order of seconds), and can also withstand the effects of masking and shifts of spatial location (Hollingworth, Richard, & Luck, 2008; Phillips, 1974).

Understanding the nature of vSTM and its limitations is important as vSTM is the interface between sensory visual perception and higher order cognitive processes, such as attention (e.g., Awh & Jonides, 2001). To assess vSTM performance, two prominent methods are typically employed: The change detection task (see e.g., Luck & Vogel, 1997; Pashler, 1988; Phillips, 1974; Phillips & Baddeley, 1971; Purdy, Eimann, & Cross Jr. 1980; Vogel, Woodman, & Luck, 2001) and the continuous report task (Bays, Catalao, & Husain, 2009; Wilken & Ma, 2004). Whilst the change detection task can be analysed using (relatively) simple signal detection methods, analysis of continuous report tasks relies on fitting probabilistic mixture models to participant data. The fitting of such models can be challenging to implement for researchers with limited programming and model-fitting experience.

The purpose of the current paper is to present mixtur: A package written in the statistical programming language R that allows users with minimal programming experience to implement the two-component mixture model of Zhang and Luck (2008) and the three-component mixture model of Bays et al., (2009). The package allows users to fit the models to participant data, but also allows for the simulation of artificial data from these models. The mixtur package provides utility functions for analysing and plotting behavioural data together with the model outcomes.

The structure of the paper is as follows. We first briefly describe the continuous report task, before presenting an overview of the two-component model of Zhang and Luck (2008) and the three-component model of Bays et al., (2009). We then present mixtur. We begin by providing an overview of all of the main functions in the package: We show how to generate model-free summary statistics of behavioural data, how to fit the mixture models to behavioural data (including formal model comparison techniques), how to plot fits of the models to behavioural data, and how to simulate data from the models. In a final section, we present a series of simulations with the aim of exploring the performance of the mixture models (e.g., parameter recovery, parameter- and model-mimicry) as well as informing experimental design considerations (e.g., number of trials to use in an experiment). In the Discussion, we describe how mixtur can also be used to fit the slots and slots-plus-averaging models described by Zhang and Luck (2008), and how mixtur can be extended in future work to fit other models of visual short-term memory.

## Continuous report task

**Fig. 1:**
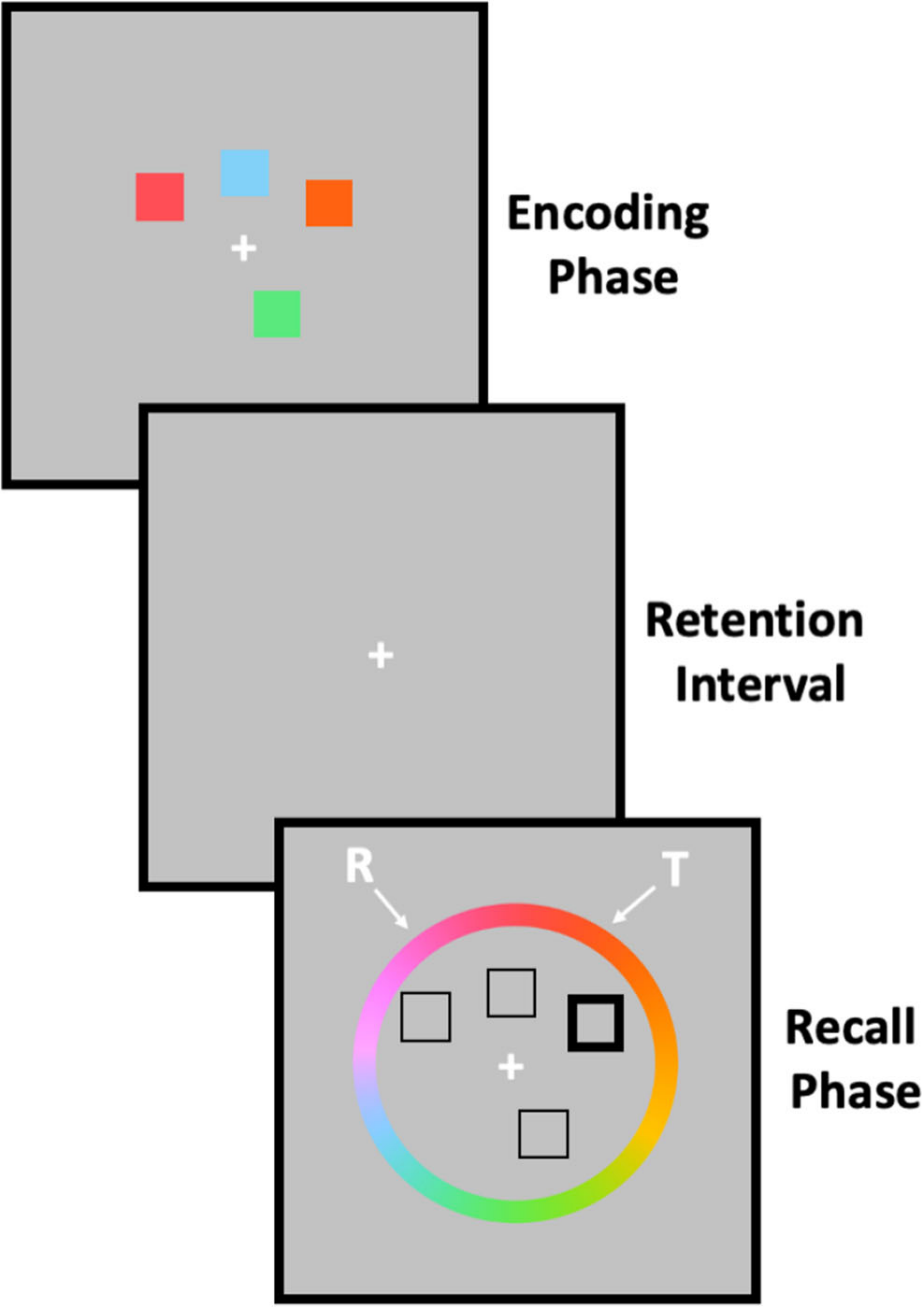
Schematic overview of a typical continuous report visual short-term memory task. R = Response value from participant. T = Target value

Unlike change detection tasks where a binary change/no-change judgement is provided, the continuous report task—popularised by Wilken and Ma (2004) but introduced by Prinzmetal, Amiri, Allen, and Edwards (1998)—is thought to provide a continuous estimate of the *precision* of the internal memory representations of presented items. In one version of the continuous report task (see for example Fig. 1) participants are presented with a stimulus display consisting of several coloured squares with the task of remembering the colours. After a variable retention interval, a probe display is then presented where the location of a specific item presented on the initial display is probed. The task of the participant is to recall and report the colour of the probed item by clicking on a colour wheel. By calculating the angular deviation between the participant’s response and the true value of the target, researchers can estimate the precision of the participant’s memory for this item: If memory for the target’s feature value is excellent, their response will be very close to the true target value (i.e., there will be minimal angular deviation); if however memory for the target’s feature value is poor, their response will be further away from the true target value (i.e., angular deviation will be high); on some trials, they may even just be guessing.

Thus, the error distributions of participant responses are thought to provide some information as to the *precision* of the internal memory representation, and can therefore be used to probe the nature of vSTM and its capacity limitations. For example, in their Experiments 7–9, Wilken and Ma (2004) used the continuous report task (using coloured stimuli in Experiment 7, and spatial frequency and Gabor patches in Experiments 8–9) and explored the effect of stimulus set size (*N* = 2, 4, 6, and 8) on memory precision. Error distributions in these experiments showed that the precision of participant responses decreased as set size increased (see also Bays et al., 2009), an observation they used as an argument against vSTM having a fixed capacity: If vSTM has a fixed capacity, it would be expected that until capacity is reached, the recall accuracy of probed items should be excellent (because all presented items will have been stored with high precision); once capacity is reached, although the items receiving a slot will still be stored with high precision, the probability of a non-stored item being probed increases, which leads to more noise in the response distributions due to increased guessing rates.

## Mixture models

Responses in the continuous report task are thought to consist of a mixture of different modes of responding: For example, in one account, it is thought that on some trials, participants are making their response based on an internal (but noisy) memory representation of the true target value; on other trials, this memory may fail (either due to recall failure or failure to encode the stimulus due to capacity limitations) and the participant merely guesses their response (e.g., Zhang & Luck, 2008). In an extension of this account, on some trials participants may also incorrectly report one of the non-probed target’s feature values (e.g., Bays et al., 2009). Researchers have therefore developed *mixture models* to quantify these different processes from behavioural data (however, see Schurgin, Wixted, & Brady, 2020 for an alternative interpretation of response error distributions in the continuous report task). Below we discuss two successful mixture models—the two-component model of Zhang and Luck (2008) and the three-component model of Bays et al., (2009)—that have been used widely to explore vSTM.

### Zhang and Luck (2008)

In the two-component model of Zhang and Luck (2008), responses in the continuous report task are a probabilistic mixture of two processes: (1) responses based on a noisy internal representation of the target’s feature value, and (2) random guessing. It is assumed that if a representation of the colour of the target item is held in memory, the reported colour value will have a propensity to be located close to the actual target value. This can be modelled as a von Mises distribution (a normal distribution for circular data) centered on the true target value with a standard deviation representing the precision of the participant’s internal representation (i.e., higher standard deviation provides poorer precision). When no representation is stored (or it is inaccessible), then no information about the colour of the probed item will be available to the participant, resulting in a random guess (which—by definition—can occur anywhere on the circle).

**Fig. 2:**
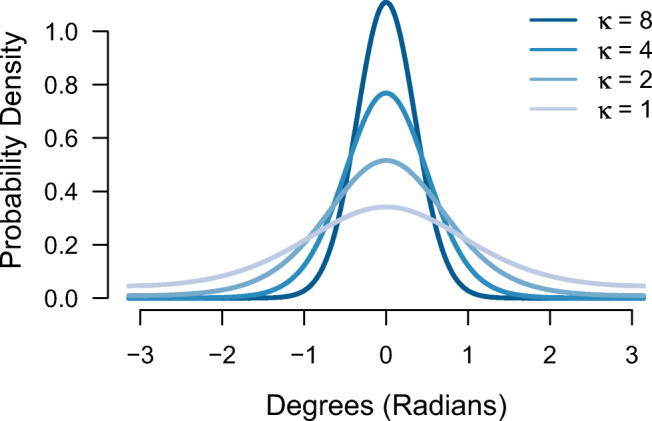
Probability density functions of different values of the von Mises concentration parameter *κ*. Higher values reflect more precise memory representations

Formally, the two-component mixture model is given by

1 $$ p(\hat\theta) = (1 - p_{u})\phi_{\kappa}(\hat\theta - \theta) + p_{u}\frac{1}{2\pi}, $$

where $\hat \theta $ is the participant’s response value (in radians), *θ* is the true target colour value (in radians), and *p*_*u*_ is the probability of giving a uniform response which captures guessing. From this, the probability of providing a target response—*p*_*t*_—is therefore given as 1 − *p*_*u*_. *ϕ*_*κ*_ represents the probability density of the von Mises distribution with mean zero and concentration parameter *κ*. Concentration parameter *κ* (kappa) is a measure of dispersion, with higher values reflecting more precise memory representations (see Fig. 2 for examples of how probability density changes with different concentration parameters *κ*).

When manipulating set size, Zhang and Luck (2008, Experiment 1) found that the parameter reflecting the probability of making a target response—*p*_*t*_—was approximately twice as high at set sizes of three when compared to set sizes of six, with no significant change in memory precision *κ*. The authors took this as evidence in support of a *slots model*: the view that a fixed and small number of item representations can be stored in memory slots with a high degree of precision. Once these slots are full (i.e., at larger set sizes), additional items are not stored at all, and if one of these non-stored items are probed at test, the participant will resort to guessing. We will return to the slots model in the General Discussion.

### Bays et al. (2009)

Accurate performance in the continuous report task does not only require accurate memory for the feature value of the target item; the participant also needs an accurate memory for which feature value was associated with which item in the stimulus display. That is, at encoding participants must bind together information regarding the target’s location as well as the target’s feature value. At test, then, errors in responding could occur due to incorrectly retrieving (with noise) a stored non-target feature value. Responses according to this *three-component* model are a probabilistic mixture of (1) responses based on a noisy internal representation of the target’s feature value; (2) responses to a non-target feature value; and (3) random guessing.

Formally, the three-component model is given by

2 $$ p(\hat\theta) = (1 - p_{u} - p_{n})\phi_{\kappa}(\hat\theta - \theta) + p_{u}\frac{1}{2\pi} + p_{n}\frac{1}{n}{\sum\limits_{i}^{n}}\phi_{\kappa}(\hat\theta - \theta_{i}^{*}) $$

where *p*_*u*_ and *κ* are as in the two-component model. The new parameter is *p*_*n*_, the probability of making a response based on a non-target feature value, with $\theta _{i}^{*}$ ($\theta _{1}^{*}, \theta _{2}^{*}, \theta _{3}^{*}, ...$) representing the feature values of the non-targets *x* (therefore *p*_*t*_ = 1 − *p*_*u*_ − *p*_*n*_). It is important to note that non-target feature values are stored with the same precision as the target feature value (i.e., *κ* is the same for target and non-target feature values) because at encoding the participant does not yet know which item will be probed.

Bays et al., (2009) used the three-component model in an experiment that manipulated set size (*N* = 1, 2, 4, and 6). The modelling showed that increasing set size resulted in significantly poorer estimates of the precision of responses and increased the frequency of non-target responses. It is theoretically important to note that Bays et al., (2009) found a reduction in precision even at set sizes of two, which is well below any putative vSTM capacity limit. Instead, this favours a resource model (e.g., Bays & Husain, 2008, Frick, 1988) whereby memory resources are evenly distributed across all items in the display,at larger set sizes, each item receives fewer resources leading to poorer memory.^1^

## Overview of mixtur

We developed mixtur to reduce the burden on researchers wishing to apply mixture modelling to continuous report data. mixtur allows users to fit both the two-component and three-component models to their data. The package allows the models to be fitted across different experimental conditions, including the common manipulation of set size as well as bespoke conditions. The fit routines will return the best-fitting parameters for each participant and each condition (if specified), allowing researchers to apply their preferred inferential analysis technique.^2^ The package will produce publication-ready plots of both behavioural data as well as plots of the model fit. The mixtur package also allows users to simulate artificial data from each model. We believe that the ability to simulate data from a model is important to explore the models in detail, as well as to help inform experimental and/or analytical design considerations (for example to calculate statistical power). In later sections of this paper, we utilise these simulation functions to explore various aspects of the models as well as establishing experiment design recommendations.

Note that we are aware of a software package MemToolbox that also implements the two- and three-component mixture models (as well as allowing modelling of data from the change detection task; Suchow, Brady, Fougnie, & Alvarez, 2013). The mixture models are also implemented in code made available to supplement (Bays et al., 2009; van den Berg, Awh, & Ma, 2014).^3^ However, these materials are written for the proprietary software MATLAB. While many researchers have access to MATLAB via institutional subscriptions, the same cannot be said for independent researchers and newer/smaller institutions without the means to supply such a subscription. Whilst there are free alternatives to MATLAB (for example Octave, Eaton, Bateman, Hauberg, & Wehbring, 2019), there are a number of MATLAB functions which have not yet been implemented, thus limiting the scope of its analytical ability. MemToolBox currently requires one of these unavailable functions, meaning that the toolbox (at present) can only be run using MATLAB. In contrast, mixtur has been written for R, a free statistical programming language. This enables everyone, regardless of circumstance, to implement mixture modelling in their research.

## Using mixtur

In this section, we provide a comprehensive overview of how to use mixtur, providing code examples for each step. mixtur is written in R (Version 4.0.2, R Core Team, 2020)^4^, a free statistical programming language. To download R, visit https://www.r-project.org/. In addition, we recommend downloading and installing R-Studio, which provides a free user interface for R https://rstudio.com/.

The mixtur package itself is hosted on the Comprehensive R Archive Network (“CRAN”). To install (and then load) the package from CRAN, run the following lines of code:The main user-facing functions available in mixtur are listed in Table 1. Users can view a help file for each function (which also include examples of the function’s usage) by typing help(FUN) into the R console, replacing FUN with the function name.

**Figure:**
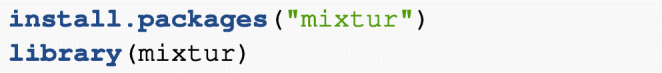

**Table 1:** A list of all of the main functions provided by the mixtur package

Function	Description
get_summary_statistics	Returns participant-level, model-free, summary statistics of response error distributions.
plot_summary_statistic	Produces a plot of the means of chosen summary statistic of response error distributions.
plot_error	Produces a plot of the response error distribution.
fit_mixtur	Fits the mixtur models to behavioural data.
plot_model_fit	Plots estimates of the model fit to observed response error distributions.
plot_model_parameters	Produces a plot of the means of the best-fitting model parameters.
simulate_mixtur	Simulates data from the mixtur models.

For more information about these functions, see the help files

mixtur ships with example data sets to allow the user to familiarise themselves with the package. All of the data sets—including a brief description of each—are listed in Table 2 and are publicly available.^5^ Users can load a particular data set by using the R function data() and can glimpse the first few rows of the data file using the head() function: 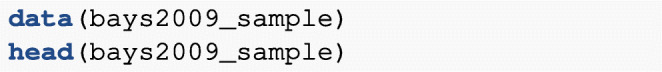



## id response target non_target_1 non_target_2 non_target_3## 1 1 -2.186 -0.002 -2.989 2.648 2.262## 2 1 -1.980 -2.498 -1.861 -1.340 -0.309## 3 1 -0.177 -2.088 -2.845 -3.102 -0.371## 4 1 1.342 1.334 2.844 1.007 -0.599## 5 1 -1.644 -2.224 3.129 2.936 1.295## 6 1 1.219 1.253 2.886 -0.924 -1.035

**Table 2:** Description of data sets shipped with the *mixtur* package

Data	Description
bays2009_full	The full data set from Bays et al., (2009) including data from 12 participants (coded in the id column). The data includes set size manipulations (1, 2, 4, and 6 items) plus a manipulation of duration of the sample array presentation (100, 500, and 2000 ms). The response, target, and non-target values are in radians (-pi to pi).
bays2009_sample	A sample of data taken from the full data from Bays et al., (2009). This data just consists of set sizes of 4 and sample array duration of 500 ms. This sample data are provided if the user wishes to interact with a simpler data structure. The response, target, and non-target values are in radians (-pi to pi).
berry_2019	The full data set from Berry, Allen, Waterman, and Logie (2019) including data from 30 participants (coded in the id column). The experiment always had a set size of three. There was an additional manipulation (coded in the condition column) indicating whether the task was completed under single-task or dual-task conditions. Note that the data has the response, target, and non-target values in degrees in the range of -180 to 180.

The Data column indicates the name of the data variable in the package

### Data structures in mixtur

mixtur has some flexibility in the data structures it can deal with, but you need to tell mixtur some things about your data so it can work with it. The data should be in long format, where rows indicate separate trials, and columns indicate trial and participant information (i.e., “tidy data”, Wickham, 2014). Typically, the data should have the columns listed in Table 3, but note that the columns do not necessarily need these names as they can be set within function calls (see examples later).

**Table 3:** Variables (as columns) expected by mixtur in data sets. The Name column shows the default names for the columns accepted by mixtur

Name	Description
id	A column indicating the participant numbers / identifiers.
response	A column providing the participant’s response for each trial. This can either be in degrees (i.e., 1-360), degrees limited to the range of 1–180 (i.e., if the experiment uses bar orientations), or radians (typically in the range 0-2pi, but could also be -pi to pi.
target	A column providing the target value. This will be used by the package to calculate response error (i.e., the deviation between the response value and the true target value). This should be in the same units as the response data.
non_target	If the experiment has set sizes greater than one, the data should include a column that provides the non-target values (one column for each non-target). If there is more than one non-target (i.e., set sizes greater than 2), each column name should begin with a common term (e.g., the “non_target” term is common to the non-target columns “non_target_1”, “non_target_2” etc.).
set_size	A column indicating the current trial’s set size (i.e., number of items to remember during the encoding phase), if your experiment manipulated this.
condition	A column indicating the current trial’s condition if your experiment had an additional manipulation (for example the duration of the sample array presentation, as in the Bays et al., 2009 data).

### Model-free summary statistics

Before discussing how to fit the mixture models, we first present how to use mixtur to obtain and then visualise model-free summary statistics—specifically, the mean absolute error, resultant vector length, precision, and bias—of the behavioural data (for an excellent overview of circular data analysis, see Cremers & Klugkist, 2018). *Mean absolute error* provides the circular mean of the absolute deviation between the response value and the true target value, with values closer to zero representing more accurate responding. The *resultant vector length* is an estimate of variability in responding, which can vary between zero and one. A resultant vector length of one means all responses matched a single value (i.e., no variability in responding) and a resultant vector length of zero means all responses were spread around the circle. Following Bays et al., (2009), the function also provides an estimate of response *precision*; this is calculated as the reciprocal of the standard deviation for circular data, subtracting from this the value expected by chance. The function also provides an estimate of response *bias*, which is the circular mean of the angular deviation between a participant’s response and the location of the true target value. Bias values close to zero indicate no clockwise or anti-clockwise bias in responses.

The function to obtain summary statistics takes the arguments presented in Table 4. We provide these arguments here in full because many other functions in mixtur share these arguments. In the following example, we take the full data from Bays et al., (2009), which is included in the mixtur package, and obtain summary statistics as a function of set size (which included set sizes of 1, 2, 4, and 6 items). Note that we need to tell mixtur the name of the column that includes information relating to the set size of each trial: 
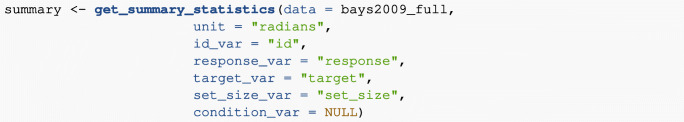

**Table 4:** Arguments that can be passed to the *get_summary_statistics()* function

Name	Description
data	A data frame containing the data that is to be plotted. See the data structure section for how this should be formatted.
unit	A character variable indicating the unit of measurement in the data. mixtur accepts units in degrees (1–360), degrees_180 capped at 180 (1–180), and radians (either 0–2pi or -pi to pi). Defaults to “degrees”.
id_var	A character variable indicating the column name that codes for participant identification. Defaults to “id”.
response_var	A character variable indicating the column name that codes for participants’ responses. Defaults to “response”.
target_var	A character variable indicating the column name that codes for the target value. Defaults to “target”.
set_size_var	If set size was manipulated, a character variable indicating the column name that codes for the set size. Defaults to NULL.
condition_var	If an additional condition was manipulated, a character indicating the column name that codes for this condition. Defaults to NULL.


## id set_size mean_absolute_error resultant_vector_length precision bias## 1 1 1 0.188 0.973 3.816 -0.023## 2 1 2 0.273 0.934 2.266 -0.042## 3 1 4 0.581 0.656 0.657 -0.005## 4 1 6 0.681 0.590 0.543 0.003## 5 2 1 0.206 0.945 2.529 0.034## 6 2 2 0.347 0.825 1.181 0.002

### Plotting

Users can obtain publication-ready plots of the model-free summary statistics, as well as a plot of the response error distribution. For plots containing multiple set sizes and experimental conditions, the colour palette of the plots can be set via the palette argument using the palettes provided in the RColorBrewer package (Neuwirth, 2014), which is installed and loaded when mixtur is loaded. To see the names of all available colour palettes, type display.brewer.all() into the R console. By default, mixtur utilises the “Dark2” palette, which is colour-blind friendly. Additionally, if the user wants the data underlying these plots, this can be achieved by setting the *return_data* argument in the respective functions to TRUE; the returned data is formatted in such a way that it is ready for inferential analysis.

#### Plotting model-free summary statistics

**Fig. 3:**
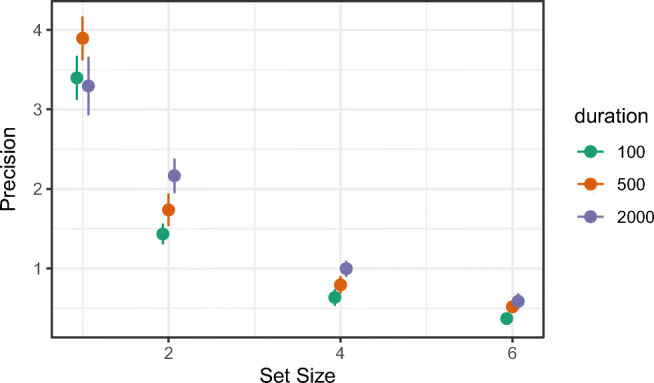
Plot of the mean response precision in the Bays et al., (2009) data, plotted as a function of set size (each plot is a different set size, with levels 1, 2, 4, and 6). *Error bars* denote ± 1 standard error around the mean

Each of the model-free summary statistics can be plotted using the plot_summary_statistic() function. The user must select which summary statistic to plot (using the *statistic* argument). By default, the plotting function returns a plot of the requested summary statistic averaged across participants, together with error bars denoting one standard error around the mean (i.e., ± 1 SE). The user can also plot by set size and/or an additional condition if this structure is present in the data. As an example, the following code plots response precision as a function of set size from the full data set of Bays et al., (2009). The plot also includes the additional manipulation of *duration* of the sample array presentation (with three levels: 100, 500, and 2000 ms); this is set by the *condition* argument. The resulting plot can be seen in Fig. 3. 
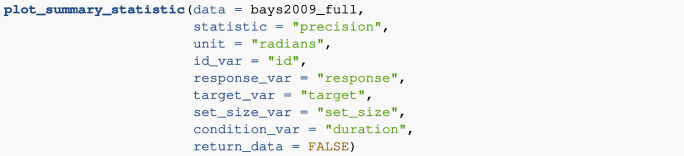

#### Plotting response error

**Fig. 4:**
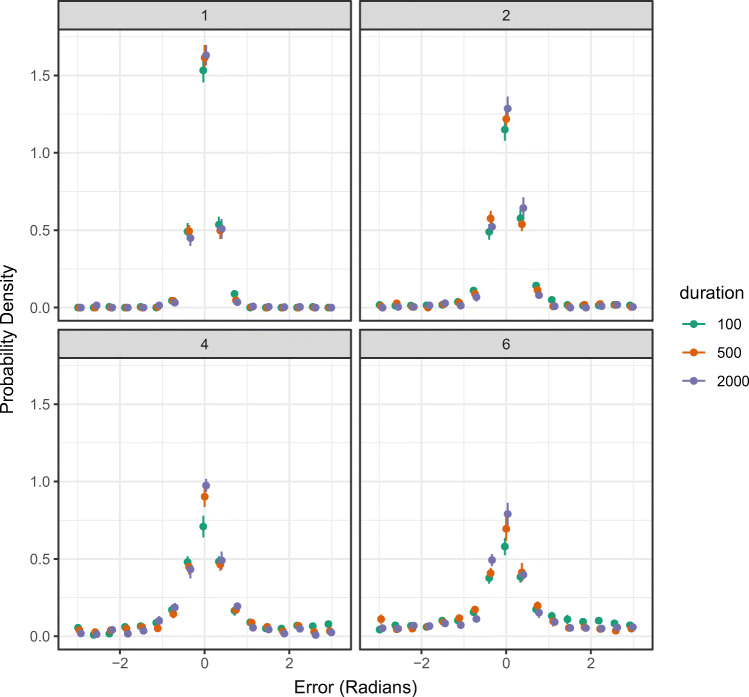
Probability density plots of participant-averaged response error (in radians) in the Bays et al., (2009) data, plotted as a function of set size (each plot is a different set size, with levels 1, 2, 4, and 6) and an additional condition manipulation of duration. *Error bars* denote ± 1 standard error around the mean

Response error refers to the angular deviation between a participant’s response and the location of the true target value (i.e., where the participant *should* have responded). By default, the plotting function returns the probability density of response error averaged across participants, together with error bars denoting one standard error around the mean (i.e., ± 1 SE). The user can also plot by set size and/or an additional condition if this structure is present in the data. In the following code, we plot response error as a function of set size and duration from the Bays et al., (2009) data. The resulting plot can be seen in Fig. 4. 
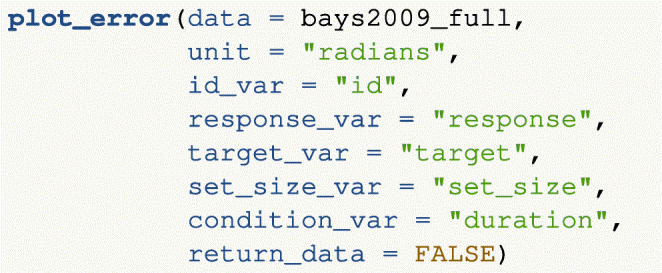

### Modelling

The main reason for developing the mixtur package was to assist researchers who wish to fit mixture models to participant data. The mixtur package can fit both the two-component mixture model of Zhang and Luck (2008) and the three-component mixture model of Bays et al., (2009), as well as the slots and slots-plus-averaging models of Zhang and Luck (2008), which we return to in the General Discussion. The models are fit to trial-level data via maximum likelihood estimation of the key parameters (see Eqs. 1 and 2) using the Nelder–Mead gradient descent algorithm to minimise the negative log-likelihood. Multiple starting values are used for each parameter to avoid local minima. Specifically, a grid of all permutations of the following parameter values are used as starting points: *κ* – 1, 10, 100; *p*_*u*_ (and *p*_*n*_ if fitting the three-component model) – 0.01, 0.1, 0.4. The model recovery simulations reported later show this fit routine has acceptable accuracy.

The models can be fit via the fit_mixtur() function. The arguments to pass to this function are similar to other functions previously discussed (see the help files by typing help(fit_mixtur) into the R console) with the addition of the *model* argument; the user should inform mixtur whether the two-component model (model = "2_component") or three-component model (model = "3_component") should be fit to the data. By setting the additional argument return_fit = TRUE, the fit routine will return the log-likelihood of model fit (which can be useful for formal model comparison; see later section).

To fit the model to individual participant data, the user should inform mixtur which column in the data set codes for participant identification (via the *id* argument). As with the plotting functions, users can inform mixtur whether there was a set-size manipulation and/or an additional condition manipulation; if so, by passing the column names to the respective arguments the package will fit the model to these independent variables.

#### Two-component model

The parameters estimated when fitting the two-component model are *κ* (kappa, the concentration parameter of the von Mises distribution) and *p*_*u*_ (the probability of a uniform response); the probability of a target response (*p*_*t*_) is calculated as 1 − *p*_*u*_. The following code shows how to fit the two-component model to data from Bays et al., (2009), fitting the model to individual participant data and the *set-size* manipulation (and ignoring the *duration* manipulation). The function returns the best-fitting parameters per participant per set size. Note that in this example (in contrast to the previous examples), it is best to save the results of the function call to a variable so that it can be accessed later (here we save the function results to the variable *model_fit_2*): 
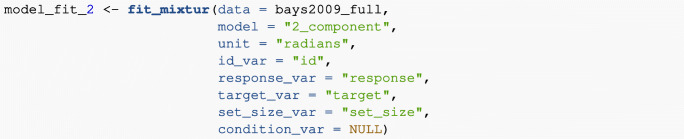

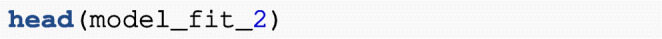



## id kappa p_t p_u set_size## 1 1 18.358 1.000 0.000 1## 2 2 16.359 0.983 0.017 1## 3 3 15.692 1.000 0.000 1## 4 4 26.955 0.986 0.014 1## 5 5 13.831 0.976 0.024 1## 6 6 27.090 0.985 0.015 1

#### Three-component model

The three-component model extends the two-component model by also taking into account binding errors (often called “swap errors”) whereby participants make an erroneous response to one of the non-cued non items held in memory. The parameters estimated when fitting the three-component model are *κ* (the concentration parameter of the von Mises distribution), *p*_*u*_ (the probability of a uniform response); and *p*_*n*_ (the probability of a non-target response). The probability of a target response, *p*_*t*_, is calculated as 1 − *p*_*n*_ − *p*_*u*_.

To fit the three-component model to the Bays et al., (2009) data (again modelling individual participant data and the set-size manipulation) users just need to alter the model argument: 
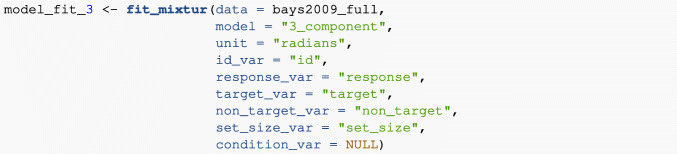

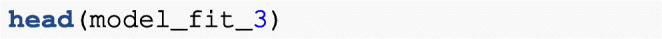



## id kappa p_t p_n p_u set_size## 1 1 18.358 1.000 0 0.000 1## 2 2 16.359 0.983 0 0.017 1## 3 3 15.692 1.000 0 0.000 1## 4 4 26.955 0.986 0 0.014 1## 5 5 13.831 0.976 0 0.024 1## 6 6 27.090 0.985 0 0.015 1

If the user wishes to obtain details of the fit routine, users can set the additional argument return_fit = TRUE. In addition to the best-fitting parameters, the function will also return the log-likelihood value, and the number of trials used in the fit routine. The function will also return Akaike’s Information Criterion (AIC) and Bayesian Information Criterion (BIC), which are estimators of prediction quality penalised by the number of model parameters. AIC and BIC can thus be used for model comparison (for both metrics, lower values suggest a superior model). AIC is given by

3 $$ \text{AIC} = - 2\widehat{LL} + 2p $$

where *LL* refers to the log-likelihood of model fit and *p* represents the number of free parameters (*p* = 2 & 3 in the two-component and three-component model, respectively). The model with the lowest AIC value is to be preferred. mixtur also provides a version of AIC corrected for small trial numbers, given by

4 $$ \text{AICc} = - 2\widehat{LL} + 2p \left( \frac{n}{n - p - 1}\right) $$

BIC is given by

5 $$ \text{BIC} = - 2\widehat{LL} + p \ln(n) $$

where *LL* and *p* are as in the AIC, *ln* is the natural logarithm and *n* is the number of trials used in the *LL* estimate.

#### Plotting model fit

**Fig. 5:**
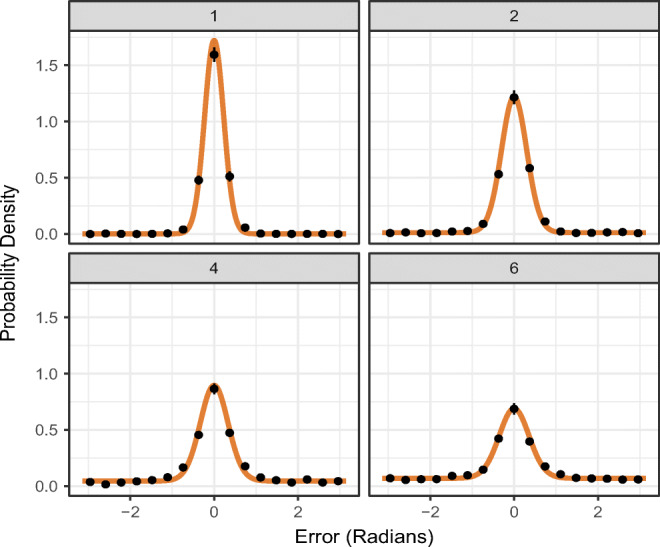
Goodness-of-fit probability density plots of model predictions of response error (shown by the *solid line*) and participant-averaged data (*points*). *Error bars* denote ± 1 standard error around the mean. Each plot shows a different set size (with levels 1, 2, 4, and 6)

**Fig. 6:**
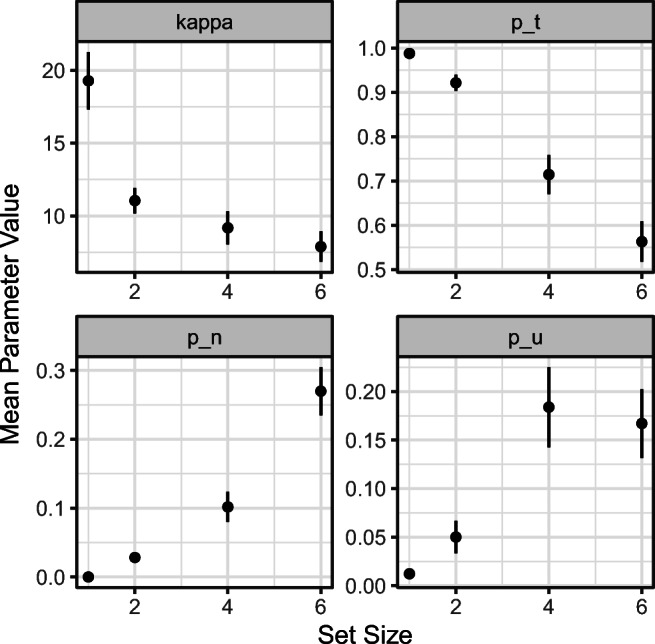
Participant-averaged estimates of best-fitting parameters from the three-component model to the Bays et al., (2009) data, plotted as a function of set size. *Error bars* denote ± 1 standard error around the mean

mixtur provides utility functions to assist the researcher in plotting model outcomes; users can plot goodness-of-fit of the model to the data, and plot participant-averaged estimates of the best-fitting parameters. Whilst the model fitting functions will return the best-fitting parameters per participant (and per set size and/or condition, if requested), it is recommended to check the goodness-of-fit of the model to the participant data. The function plot_model_fit() in mixtur will plot the model-predicted response error against the participant-averaged data.^6^ We pass to this function the variable containing the participant data, and the variable containing the model fit results (other arguments are similar to previous functions), as well as stating which model was used to fit the data. In the following example, we plot the fit of the three-component model to the Bays et al., (2009) data by set size, which produces Fig. 5: 
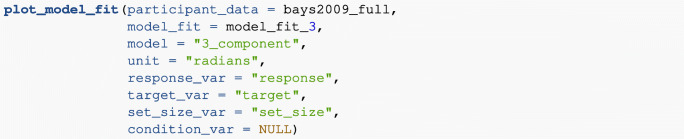

The plot_model_parameters() function can be used to plot the average parameter values across participants for each set size and condition (if manipulated) by passing to it the variable containing the model fit information and the model that was fit to the data (Fig. 6). As withprevious plotting functions, the user can set return_data = TRUE to obtain the data underlying the plot. 
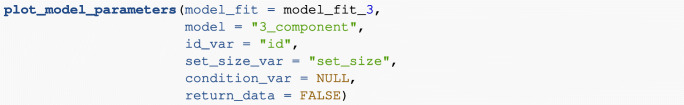

### Simulating

As well as fitting models to participant data, mixtur can also be used to simulate artificial data from both the two-component and three-component models. The critical function—simulate_mixtur()—needs to be passed as arguments the name of the model to simulate from, the parameters to use in the simulation, as well as the number of trials and the set size to use in the simulation. If simulating from the two-component model, values for *κ* and *p*_*u*_ need to be provided; if simulating from thethree-component model, a value for *p*_*n*_ must also be provided.

In the following example, we simulate 5000 trials from the two-component model with a set size of 4, *κ* = 15 and *p*_*u*_ = 0.25. The simulation returns (in radians) the target feature value, the simulated response from the model, and the non-target feature values. These non-target feature values do not influence the simulated data from the two-component model, but do influence responses for the three-component (the degree to which is set by the *p*_*n* argument when simulating from this model). 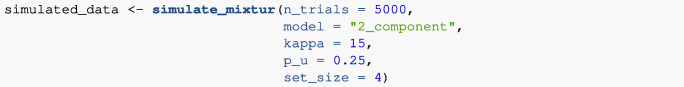

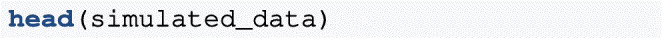



## id set_size target response non_target_1 non_target_2 non_target_3## 1 1 4 0.157 -0.178 0.541 1.222 -2.025## 2 1 4 -3.054 2.866 2.670 -0.454 0.890## 3 1 4 2.112 1.803 -1.833 -0.209 2.478## 4 1 4 -2.601 -2.342 -1.606 2.531 -0.681## 5 1 4 0.140 0.387 -2.391 2.182 -1.990## 6 1 4 1.344 1.635 -0.611 2.740 -0.244

If the user wishes to simulate multiple set sizes at once, a vector of set sizes can be passed to the set-size argument; however, if choosing this option, the user must also provide a vector of matching length to each model parameter argument to use for each set size. For example, the following code simulates data from the two-component model for set sizes of 1, 2, 4, and 6, with *p*_*u*_ increasing with set size: 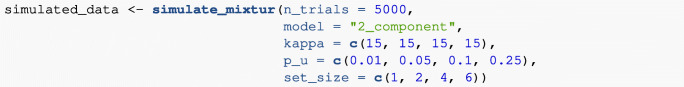

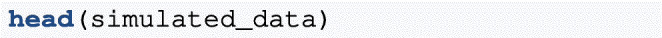



## id set_size target response non_target_1 non_target_2 non_target_3## 1 1 1 1.955 1.659 NA NA NA## 2 1 1 -0.611 -0.848 NA NA NA## 3 1 6 -0.890 -0.869 -1.466 1.955 -3.107## 4 1 6 -1.344 -1.037 -2.094 -0.768 2.059## 5 1 6 -1.658 -1.963 0.279 -2.810 2.705## 6 1 2 1.850 1.632 -2.426 NA NA## non_target_4 non_target_5## 1 NA NA## 2 NA NA## 3 0.070 0.855## 4 -2.985 0.908## 5 3.089 -0.349## 6 NA NA

## Simulations

In this section, we present the result of simulations conducted to assess some fundamental properties of the model and the fitting procedure to establish recommendations for the design of experiments that wish to utilise these mixture models.

### Simulation 1: parameter recovery

In the first simulation, we were interested in the accuracy of the fitting routine in estimating model parameters. To explore this issue, we conducted a parameter recovery simulation, whereby we simulate data from synthetic participants with known parameter values, and then fit the model to these synthetic participants; if model fitting is accurate, the fitting routine should recover the parameters used to generate the data. In this simulation, we also explored the effect of the number of trials on the accuracy of parameter recovery to establish recommendations for researchers designing experiments to assess these parameters in participants.

We simulated the two-component and three-component model separately. For each simulation, we generated data from 500 synthetic participants; each synthetic participant’s data set consisted of *N* simulated trials, where *N* varied across simulations with the levels 20, 50, 100, 200, 500, and 1000. The parameters used to simulate each participant’s data were randomly generated from a uniform distribution for each model parameter covering a range of plausible values. For the two-component model, the range of possible parameter values was *κ* = 1–16 and *p*_*u*_ = 0.00–0.40; for the three-component model it was *κ* = 1–16, *p*_*u*_ = 0.00–0.40, and *p*_*n*_ = 0.00–0.14.

**Fig. 7:**
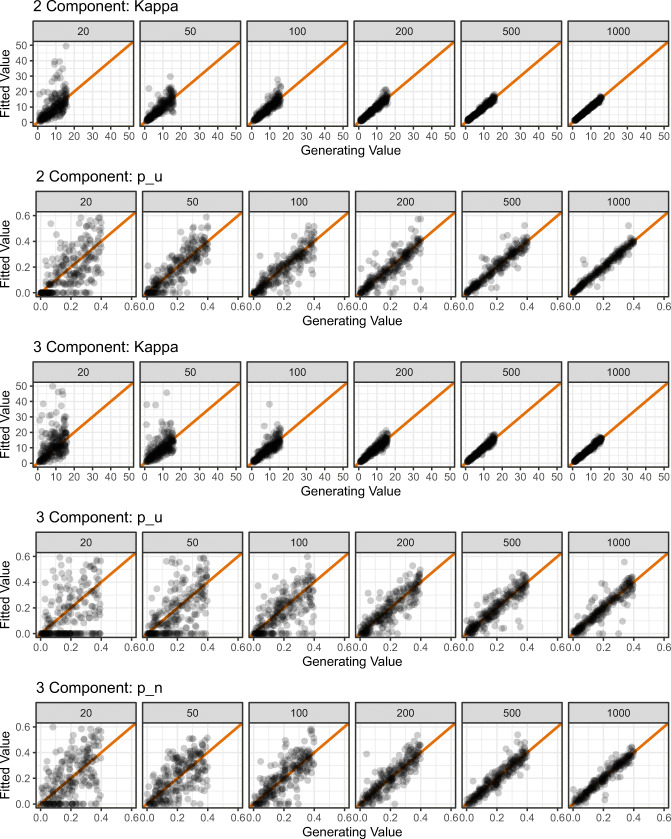
Results of parameter recovery simulations for the two-component model parameters (rows 1 and 2) and the three-component model parameters (rows 3–5). *Points* represent the best-fitting parameter values (*y*-axis) plotted against generating parameter values (*x*-axis) for 500 simulated participants. *Column titles* show the number of trials used in each simulation. The *diagonal line* represents perfect correspondence between generated and fitted parameter values

To assess the quality of parameter recovery, we calculated the product–moment correlation between generated and recovered parameter values. Following White, Servant, and Logan (2018), parameter recovery was considered poor if *r* < .5, fair if .5 < *r* < .75, good if .75 < *r* < .9, and excellent for *r* > .9. The results of the simulation can be seen in Fig. 7, and the correlation values in Appendix B.

For the two-component model, parameter recovery was good with as few as 50 trials (*r*_*κ*_ = .82, *r*_*pu*_ = .84); for excellent recovery, 200 trials were required for *κ*, and 500 trials were required for *p*_*u*_. For the three-component model, 200 trials were required before all parameters were recovered to a good level (*r*_*κ*_ = .93, *r*_*pu*_ = .86, *r*_*pn*_ = .88). The results of this simulation suggest that at least 200 trials are required (per set size) for good-to-excellent parameter estimation.

### Simulation 2: parameter trade-offs

In the next simulation, we were interested in exploring whether model parameters trade-off against each other during the fit routine. That is, if we systematically vary one parameter across a range of values when generating simulated data and hold all other model parameters constant, then the fit routine should show a selective change in the parameter that was varied and no change in the constant parameters. If we find that non-varied parameters also change during the fit-routine, this would suggest that there is some degree of trade-off in the model parameters.

We explored this in another parameter recovery study; the two-component and three-component model were explored in separate simulations. For each model, we conducted a separate parameter recovery simulation per model parameter where we systematically varied that parameter whilst holding other parameters at a default constant value; 50 parameter values were explored for the varying parameter. The default parameter values^7^ were *κ* = 8 and *p*_*u*_ = 0.10 for the two-component model, and *κ* = 8, *p*_*u*_ = 0.10, and *p*_*n*_ = 0.15 for the three-component model. When varied, 50 equally spaced parameter values were selected from the following ranges: *κ* = 4–12; *p*_*u*_ = 0.05–0.80; and *p*_*n*_ = 0.05–0.80. For each combination of parameter values, 500 trials were simulated. The relevant model was then fit to this data set, and the best-fitting parameters were compared against the generating parameter values. This process was repeated 500 times for each combination of parameter value.

**Fig. 8:**
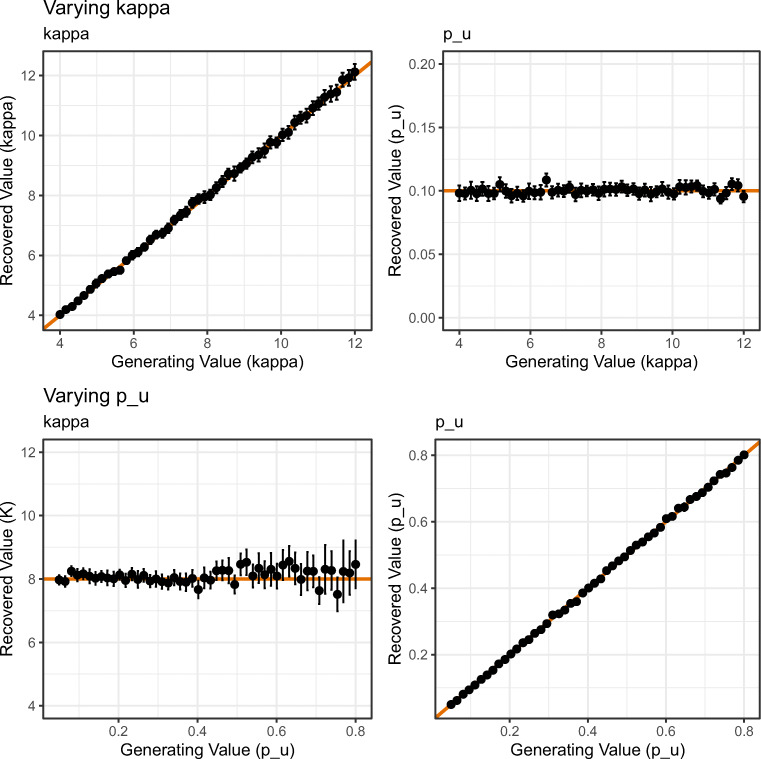
Results of the parameter recovery of the two-component model in Simulation 2. *Points* represent the mean of 500 simulations for each combination of parameter value (*error bars* denote 95% confidence intervals). The *solid line* represents perfect correspondence between generated and fitted parameter values

The results for the two-component model can be seen in Fig. 8. The first row shows parameter recovery estimates for *κ* and *p*_*u*_ when *κ* was systematically varied. The second row shows parameter recovery estimates for *κ* and *p*_*u*_ when *p*_*u*_ was systematically varied. The solid line shows the generating parameter value in each plot. As can be seen in the top row of Fig. 8, when *κ* was systematically varied, the parameter recovery correctly showed a corresponding and selective increase in the *κ* parameter; had *p*_*u*_ varied as *κ* was varied this would suggest a degree of parameter trade-off. This however was not observed. There was also little evidence for parameter trade-offs when *p*_*u*_ was systematically varied (lower row of Fig. 8).

However, when *p*_*u*_ was varied to values larger than 0.6, the accuracy of estimating the *κ* parameter became less stable. The mean absolute deviation of parameter estimates from the generating value of 8 was below 0.898 when *p*_*u*_ was below the 60th percentile of all *p*_*u*_ values explored (equating to *p*_*u*_=.46), but increased above 1.22 when *p*_*u*_ was above the 70th percentile (equating to *p*_*u*_=.54). This suggests that when high degrees of guessing are present in the data set, estimation of *κ* becomes less accurate.

**Fig. 9:**
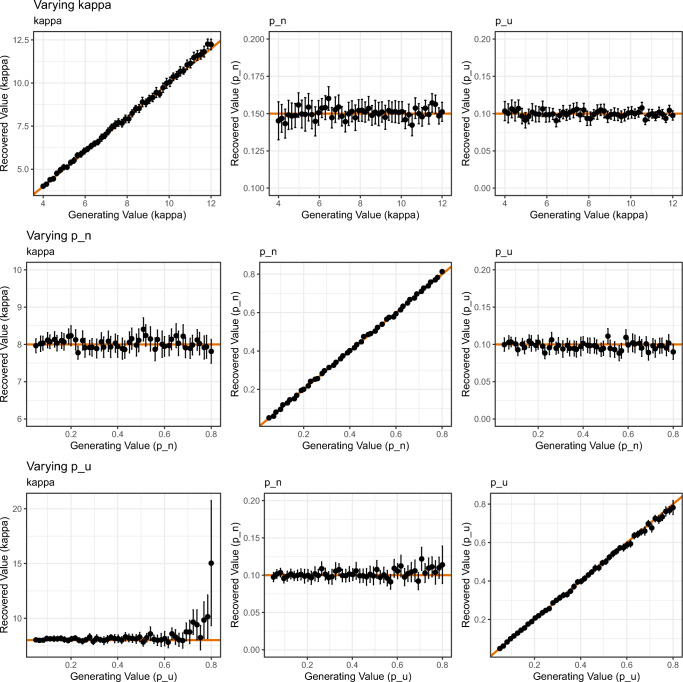
Results of the parameter recovery of the three-component model in Simulation 2. *Points* represent the mean of 500 simulations for each combination of parameter value (*error bars* denote 95% confidence intervals). The *solid line* represents perfect correspondence between generated and fitted parameter values

The picture was similar for the results of the three-component model, as seen in Fig. 9. When *κ* was varied (top row), variation in recovered parameter values was limited to *κ*, where recovery was excellent; neither *p*_*n*_ nor *p*_*u*_ systematically varied. Likewise, variation in recovered parameter values was limited to *p*_*n*_ when *p*_*n*_ was varied (middle row), and variation in recovered values was limited to *p*_*u*_ when *p*_*u*_ was varied (middle row). However, we observed the same pattern as for the two-component model: The mean absolute deviation of parameter estimates from the generating value of 8 was below 0.94 when *p*_*u*_ was below the 50th percentile of all *p*_*u*_ values explored (equating to *p*_*u*_=.39), but increased above 1.10 when *p*_*u*_ was above the 60th percentile (equating to *p*_*u*_=.46).

### Simulation 3: model recovery

Simulations 1–2 together suggest that parameter recovery of both the two-component and three-component model is very good. In Simulation 3, we were interested in exploring the extent to which the models mimic each other. As the three-component model is an extension of the two-component model, it can fit any data set equally as well as the two-component model (for example, when the *p*_*n*_ parameter is set at zero, it behaves identically to the two-component model). Theoretically, this additional flexibility is thought essential to account for binding (or swap) errors, where on some trials participants erroneously recall the feature value of one of the non-probed targets (Bays et al., 2009). As the additional flexibility of the three-component model is a theoretical stance, some researchers may be interested in assessing whether the two-component or three-component model best accounts for empirical data. The earlier section describing model fitting outlined how mixtur is able to conduct model competition using Akaike’s Information Criterion (AIC) and Bayesian Information Criterion (BIC), both of which jointly account for goodness-of-fit and model complexity.

However, if researchers are engaged in a program of work formally comparing the fits of the two-component and three-component model, it would be important to know the extent to which the two models mimic each other. That is, if the two-component model were the “true” generating process in participant data, the two-component model should win the formal model competition (likewise if the “true” generating process were the three-component model, this model should win the formal model comparison).

We explored this question via a model recovery simulation: We simulated data from a “true” model (e.g., both the two-component model and the three-component model, in separate simulations), and then conducted formal model comparison on this data set by fitting both the two-component and the three-component model using both AIC and BIC. Repeating this process many times, we were then able to quantify the percentage of fits where the true generating model was accurately recovered.

**Fig. 10:**
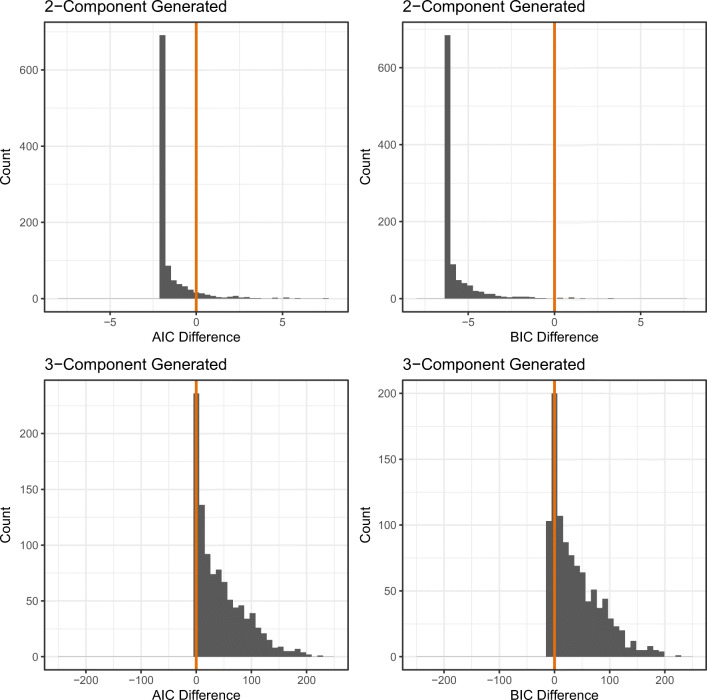
Histograms of the AIC and BIC difference scores for the model recovery simulation (Simulation 3). The *upper row* shows the AIC and BIC difference scores when the two-component model was the true generating model. The *lower row* shows the AIC and BIC difference scores when the three-component model was the true generating model. Values greater than zero (marked by the *vertical line* in each plot) indicate a better fit for the three-component model, and values below zero a better fit for the two-component model

For each model, we simulated 1000 data sets, with each data set comprising 500 trials. To each data set, we then fit both the two-component and three-component model to the data. We then calculated the difference in AIC between the two-component model and three-component model fit (where *AIC*_*difference*_ = *AIC*_2*component*_ − *AIC*_3*component*_), and did the same for BIC, with values above zero indicating a better fit for the three-component model and values below zero indicating a better fit for the two-component model. Fig. 10 shows histograms of the AIC and BIC difference scores across these simulations.

As can be seen, when the two-component model was the true generating model, both the AIC and BIC were able to correctly recover this model in the majority of simulations. The two-component model was correctly recovered in 92.8% of the simulations via AIC, and 99.7% of the simulations via BIC. When the three-component model was the true generating model, AIC recovered the correct model in 87.8% of the simulations, and BIC recovered the correct model in 77.6% of the simulations. Although both AIC and BIC correctly recovered the generating models in the majority of simulations, AIC performed (on average) better than BIC, likely due to BIC’s over-penalisation of model complexity, a result also reported by Donkin, Nosfsky, Gold, and Shiffrin (2015) and van den Berg, Awh, and Ma (2014)using similar mixture models.

### Simulation 4: effect of set size

In Simulation 4, we explored the effect of stimulus display set size on the ability of the models to accurately recover true generating parameters. The mathematics of the three-component model takes into account the feature values of non-probed items when calculating the proportion of binding errors (captured by the *p*_*n*_ parameter) so it is plausible that the number of items presented to participants might influence the accuracy of estimating this (and other) parameters.

All of the simulations reported so far were conducted using the default set size in the simulate_mixtur() function of 4. These simulations have shown that—with a good number of trials—the model parameters can be accurately recovered. In Simulation 4, we varied the set size used in simulating artificial data, exploring set size values of 2, 3, 4, 6, and 8. As the two-component model does not consider non-probed items in its mathematics, we did not include this model in the simulations.

**Fig. 11:**
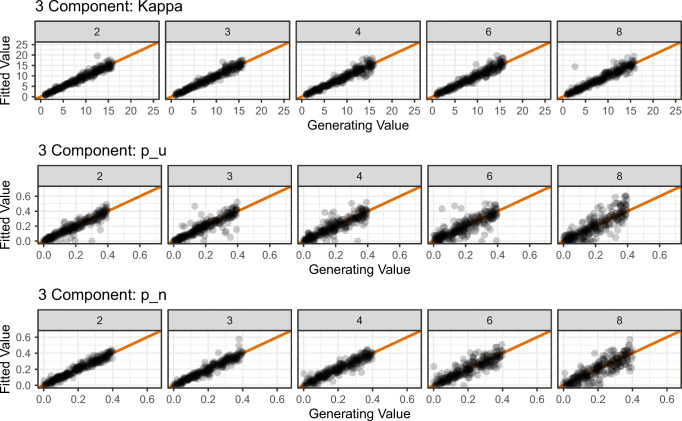
Results of parameter recovery simulations for the parameters of the three-component model as a function of the set size of stimulus display (i.e., number of memoranda). *Points* represent the best-fitting parameter values (*y*-axis) plotted against generating parameter values (*x*-axis) for 500 simulated participants. Column titles show the set size (i.e., number of memoranda) used in each simulation. The *diagonal line* represents perfect correspondence between generated and fitted parameter values

We simulated 500 trials for each level of set size from the three-component model; the generating parameter values were randomly selected using the same process as in Simulation 1. We then fit the three-component model to the simulated data and stored the best-fitting parameter values. This process was then repeated for 500 simulations. The recovered parameter values are plotted against the generated values as a function of set size in Fig. 11. As with Simulation 1, we calculated product–moment correlations to assess adequacy of parameter recovery, which are reported in Appendix B.

As can be seen in the upper row of Fig. 11, recovery of *κ* was excellent throughout all set sizes (all *rs* > .95). Although the parameter recovery for parameters *p*_*u*_ and *p*_*n*_ was always good (i.e., all *r* > .75, there was a slight decline in parameter recovery adequacy with increasing set size for both *p*_*u*_ (*r* = .933 at set size 2, *r* = .850 at set size 8) and *p*_*n*_ (*r* = .972 at set size 2, *r* = .842 at set size 8).

### Simulation 5: memoranda similarity

In Simulation 5, we explored the effect of the distance in feature space between memoranda on parameter recovery adequacy. In continuous report studies, researchers often put constraints on the minimum difference between memoranda to ensure participants can discriminate between memoranda. For example, colours may be randomly selected from a (360 degree) colour wheel with the constraint that each stimulus feature value must be at least 40 degrees away from any other feature value. Whilst this is useful for enhancing the discriminability of memoranda, it is unclear whether the spacing of non-probed item feature values impacts the ability of the three-component model to differentiate between *p*_*n*_ errors and *p*_*u*_ errors, leading to a mis-specification of errors (and inaccurate parameter estimates). That is, whilst some minimum degree of separation is good for participants to discriminate between items, does this value affect the accuracy of the model’s parameter estimation?

We explored this question in a parameter recovery simulation with a set size of 4. Differences between each feature value across each of the four items on each trial was fixed at a particular value: Either 5, 10, 15, 20, 30, or 40 degrees difference. For example, with a difference of 5 degrees, the feature values could be 17, 22, 27, and 32 degrees. For each level of separation explored, 500 trials were simulated (by randomly selecting generating parameters using the same process as in Simulation 1) and the model was fit to this data set. This process was repeated 500 times. Note that as the two-component model does not consider non-probed feature values, we only simulated the three-component model.

**Fig. 12:**
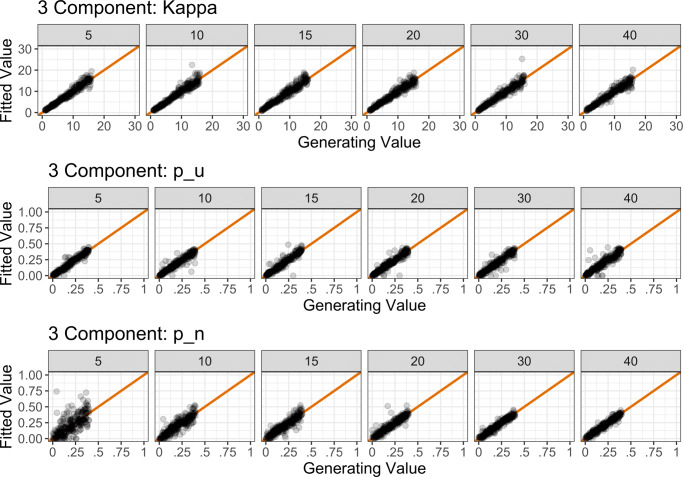
Results of the memoranda similarity simulation for the three-component model. In all plots, the points represent the mean recovered parameter values across 500 simulations for each level of degree separation. *Error bars* denote 95% confidence intervals. The *horizontal line* in each plot represents each parameter’s true generating value. **A** Recovery results for *κ*. **B** Recovery results for *p*_*u*_. **C** Recovery results for *p*_*n*_

The results of the simulation can be seen in Fig. 12. As can be seen, across a the whole range of memorandum similarity explored, parameter recovery was excellent for both *κ* and *p*_*u*_ (minimum *r*=.914). However, for *p*_*n*_, parameter recovery was only fair (*r*=.673) for a separation of 5 degrees, and good (*r*=.883) for a separation of 10 degrees, becoming excellent (*r*=.906) with 15 degrees of separation. Together, these results suggest that—whilst some degree of separation is advised to ensure participants can discriminate between memoranda—the minimum value chosen for item similarity does not impact on parameter estimation quality for *κ* or *p*_*u*_, but at least 15 degrees of separation is required to estimate *p*_*n*_ to an excellent level.

## General discussion

The application of mixture modelling to continuous report visual short-term memory data has advanced theorising of vSTM and its capacity limitations (Bays et al., 2009; Zhang & Luck, 2008). mixtur provides the researcher with a set of utility functions that aid the design, analysis, and modelling of continuous report visual short-term memory studies using the two-component and three-component models. In addition to providing a comprehensive overview of how to use mixtur, we also conducted simulation studies to develop some clear recommendations for researchers wishing to apply these models to their data. We outline these below.

### Design recommendations

In Simulation 1, we found that for the two-component model parameter recovery for *κ* and *p*_*u*_ was good with as few as 50 trials, but 200 trials were required for *κ*’s recovery to become excellent, and 500 for *p*_*u*_. For the three-component model, 200 trials were required for very good parameter recovery. Given these outcomes, we recommend that at least 200 trials should be used per cell of the experimental design (i.e., per set size and/or experimental condition). Oberauer, Stoneking, Wabersich, and Lin (2017) demonstrated via simulations using the two- and three-component models (as well as the interference model of Oberauer & Lin, 2017) that low trial numbers can be compensated for by larger participant sample sizes when using hierarchical Bayesian modelling methods, leading to improved parameter estimation. These methods are, however, not currently implemented in mixtur.

In Simulation 2, we showed that generally the parameters did not trade-off against each other (i.e., systematic variation of one parameter led to selective changes in estimates of just that parameter). However, when *p*_*u*_ was varied above around 0.5, accurate recovery of the concentration parameter *κ* became unstable; this was true for both the two-component model and three-component model. Thus our recommendation is that researchers should be cautious about interpreting *κ* for a participant whose *p*_*u*_ parameter is above 0.5. One might even consider using *p*_*u*_ > 0.5 as an a priori exclusion rule for participant data. Other studies have highlighted poor recovery of precision parameters when guessing rates are high. For example, Sutterer and Awh (2016) conducted a simulation wherein data was generated based on the two-component model of Zhang and Luck (2008). The authors observed a systematic *over- estimation* of precision when the probability an item was held in memory was below 40% (i.e., *p*_*u*_ greater than 0.6). However, our recovery of the two-component model found increased variability in *κ* when *p*_*u*_ was greater than 0.6 rather than a systematic over-estimation. It is also important to note that the Bayesian hierarchical approach demonstrated by Oberauer et al., (2017) minimises this estimation bias.

The competitive model recovery simulation (Simulation 3) showed that when the data was generated by the two-component model, generally this model would fit the data better than the three-component model, and when the data was generated by the three-component model, generally this model would fit the data better than the two-component model. This suggests that these models can be used to determine with some accuracy whether the data were generated by a two-component process (i.e., a mixture of responses to the target value plus random guessing) or a three-component process (i.e., a mixture of responses to the target value, responses to non-target values, and random guessing). There is a limit, though, on how much “better” the two-component model can ever fit the data compared to the three-component model even when it is the true generating process. This is because the three-component model can always fit data at least as well as the two-component model because when *p*_*n*_ is set to zero, the three-component model becomes the two-component model. Therefore, according to Eq. 3, in cases where both models fit the data equally well, the deciding factor is the model complexity; according to Eq. 3, then, when fit is identical (e.g., if LL is factored out of the equation) the AIC difference becomes:

6 $$ \begin{array}{lll} AIC_{difference} &= & AIC_{two-component} - AIC_{three-component}\\ &=& 2k_{two-component} - 2k_{three-component})\\ &=& (2 * 2) - (2 * 3 )\\ &=& -2 \end{array} $$

Thus, the maximum AIC difference that can ever be achieved in support of the two-component model—even when it is the “true” generating model—is an AIC difference of -2. A similar outcome is true for the BIC statistic, where—given a fixed number of trials—the maximum BIC difference in support

of the two-component model is a constant value. In contrast, because the three-component model *can* fit data much better than the two-component model when it is the true generating model, there is no upper limit on the AIC or BIC difference in model fit. Thus researchers should keep this asymmetry in mind if engaging with model competition studies.

### Slots models

The main motivation for mixtur was to provide an implementation of the popular two- and three-component mixture models. These models are so-called measurement models in the sense that they quantify key parameters of interest to explain response error (i.e., memory precision, guessing, etc.). However, the models do not provide an explanation for these errors. This provides some flexibility as to the type of experimental data that mixtur can be used for. For example, versions of these mixture models have also been used to explore accessibility and precision of episodic memory representations (Berens, Richards, & Horner, 2020; Richter, Cooper, Bays, & Simons, 2016).

Explanatory models on the other hand do provide mechanistic explanations for observed errors. mixtur can be extended to accommodate such models. Indeed, two explanatory models—the *slots* model and the *slots-plus-averaging* models of Zhang and Luck (2008)—are already implemented in mixtur. The slots model assumes that visual short-term memory consists of a fixed number of slots; each slot can store one item in the display with high resolution. However, if set size is larger than the number of slots, some items in the display will not be encoded, and if one of these non-encoded items are probed at test the participant will resort to guessing. In this model, the number of slots is given by parameter *K*, and the precision of the representation of items stored in the slots is given by parameter *κ* (kappa). This is a re-parameterisation of the two-component model, providing an explanation for the guessing rate (i.e., *K*) rather than statistically estimating its consequences (i.e., *p*_*u*_). An extension to the slots model is the slots-plus-averaging model. This model is similar to the slots model in that it is assumed vSTM consists of a fixed number of slots (again given by *K*). However, when *K* is larger than the set size, items can be stored in more than one slot (until all slots are full); at test, the average value in each slot is available for report. This leads to better memory for items that are stored in multiple slots. This model provides a good account of the set-size effect (decreasing precision with increasing set size). We provide a full overview of how to fit and simulate these models in Appendix C; we also repeat the design simulations using these models (the outcome of which do not alter our recommendations in this section).

### Future directions

Although the slots and slots-plus-averaging models are implemented in mixtur, there are others that are currently not implemented that could be the source of future developments of mixtur. One example is the non-parametric version of the three-component model developed in Bays (2016): Whilst the models discussed in the current paper assume noise in vSTM representations is captured by a von Mises distribution, the model presented by Bays (2016) does not require this assumption. Bays (2016) revealed that by relaxing this strong assumption, the non-parametric model discovered that estimates of the frequency of binding errors (i.e., *p*_*n*_) are much more frequent than suggested by the parametric models provided in mixtur. In the neural resource model of Bays (2014; see also Schneegans et al., 2020), feature values are encoded into vSTM via activity in simulated neural population codes, with separate neurons tuned to prefer particular feature values; errors at recall are explained by the noise associated with these population codes.

Another model is the interference model of Oberauer and Lin (2017). The model explains vSTM errors as arising from interference in memory from three sources: (1) location-based cuing triggering activation of non-target feature values; (2) residual activation from non-target feature values presented at encoding; and (3) random guessing. The interference model appears a very promising model as it builds on the successes of interference models in explaining errors in verbal working memory (e.g., Oberauer, Lewandowsky, Farrell, Jarrold, & Greaves, 2012), thus providing a unifying account of memory.

Models wherein precision is variable across items and trials (see for example van den Berg et al., 2012), have been shown to better account for data obtained from continuous report tasks (as well as change localisation) compared to slot models. Indeed, in a factorial comparison of multiple models of vSTM, van den Berg et al., (2014) showed that the underlying nature of precision was variable across both items and trials, as opposed to being fixed (e.g., Zhang & Luck, 2008) or equal (Wilken & Ma, 2004), with increasing set sizes resulting in reductions of precision. However, this comparison also revealed that a model wherein the number of items remembered is fixed or Poisson-distributed performs better than a model wherein all items are remembered. While this may suggest a slot model may be best suited to account for vSTM performance, van den Berg et al. showed that the number of remembered items is underestimated by models assuming a fixed number of remembered items. The authors highlight that such models do not account for variability or non-target responses, meaning that responses provided on the basis of a low-precision or non-target representation are erroneously considered as items not held in memory by these models. As such, a variable precision model wherein the number of remembered items is fixed or Poisson-distributed appears to be a more appropriate model than a simple fixed precision slot model.

A further model that could be implemented in the mixtur package is the target confusability competition (TCC) model by Schurgin et al., (2020), which proposes that errors in the continuous report task do not arise from a mixture of different memory states (e.g., noisy memory response or guessing), but rather arise naturally from the psychophysical similarity (and hence, potential confusability) of stimuli used in continuous report tasks (e.g., colours on a colour wheel). The model assumes that responses are based on a noisy familiarity signal, with colours close to the true target feature value receiving high levels of familiarity (and hence, an increased probability of being chosen as the response), however, the strength of familiarity falls exponentially as distance from the target value increases, meaning values further from the target value receive less familiarity and hence are associated with a reduced probability of being selected. The model has been shown to parsimoniously account for performance across a wide array of manipulations (e.g., set size, sample duration) and stimulus spaces (e.g., colours and faces). At present, however, the TCC model does not account for binding errors, which appear prevalent in continuous report tasks (Bays et al., 2009; Oberauer and Lin, 2017) as implemented in the three-component mixture model.

### Conclusion

We hope that mixtur will be a useful and accessible tool for anyone who wishes to apply mixture modelling to their research, specifically those who have minimal programming knowledge or limited/no access to proprietary software. Furthermore, we hope that the recommendations discussed above will not only assist those currently utilising mixture modelling, but also provide a good basis for anyone who may wish to do so in the future.

## Open practices statement

The source code for mixtur is hosted at the first author’s GitHub site https://github.com/JimGrange/mixtur where users can also post bug reports. The code and associated data for the simulations can be found at https://osf.io/yn9sf/.

## Figures and Tables

**Fig. 13:**
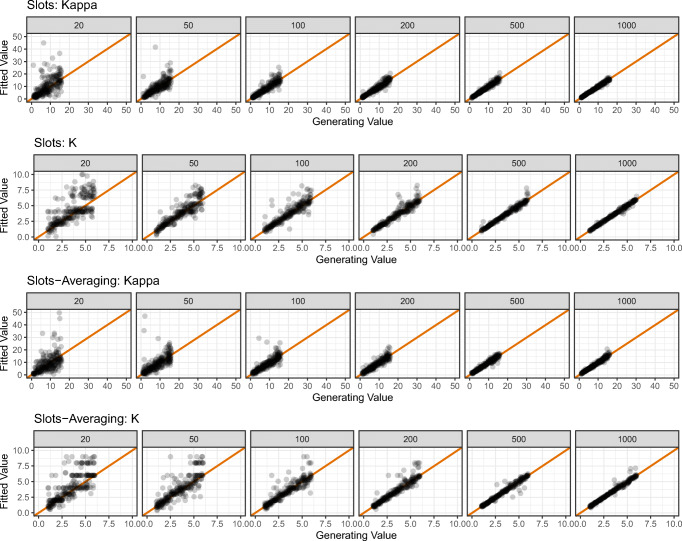
Results of parameter recovery simulations for the slots model parameters (rows 1 and 2) and the slots-plus-averaging parameters (rows 3 and 4). *Points* represent the best-fitting parameter values (*y*-axis) plotted against generating parameter values (*x*-axis) for 500 simulated participants. Column titles show the number of trials used in each simulation. The *diagonal line* represents perfect correspondence between generated and fitted parameter values

**Fig. 14:**
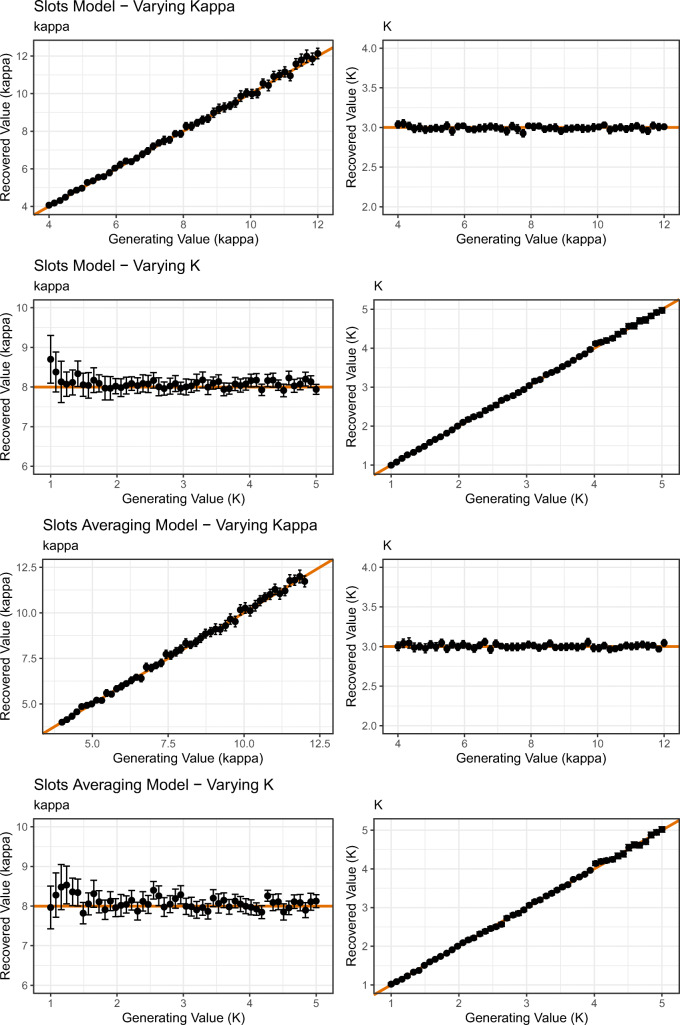
Results of the parameter trade-off simulation for the slots model and the slots-plus-averaging model. *Points* represent the mean of 500 simulations for each combination of parameter value (*error bars* denote 95% confidence intervals). The *solid line* represents perfect correspondence between generated and fitted parameter values

**Fig. 15:**
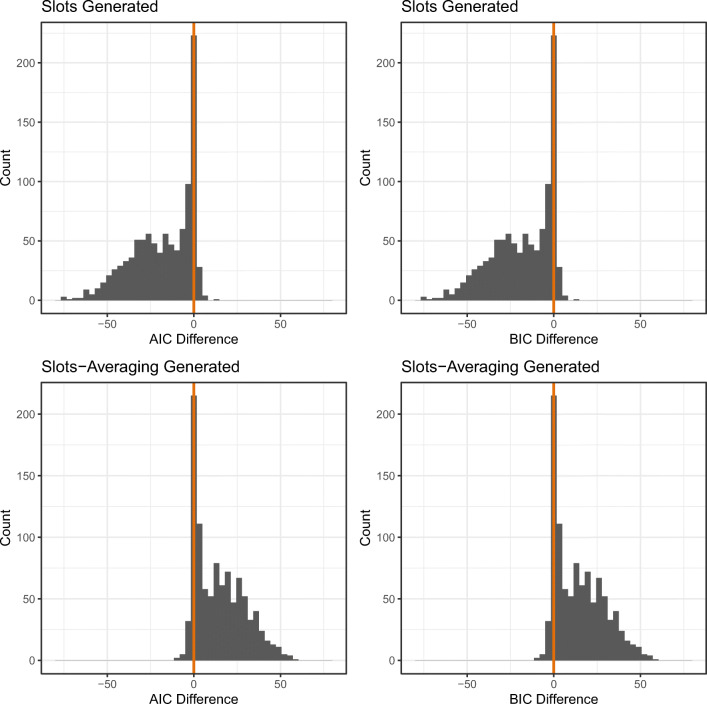
Histograms of the AIC and BIC difference scores for the model recovery simulation for the slots and the slots-plus-averaging models. The *upper row* shows the AIC and BIC difference scores when the slots model was the true generating model. The *lower row* shows the AIC and BIC difference scores when the slots-plus-averaging model was the true generating model. Values greater than zero (*marked by the vertical line in each plot*) indicate a better fit for the slots-plus-averaging model, and values below zero a better fit for the slots model

**Fig. 16:**
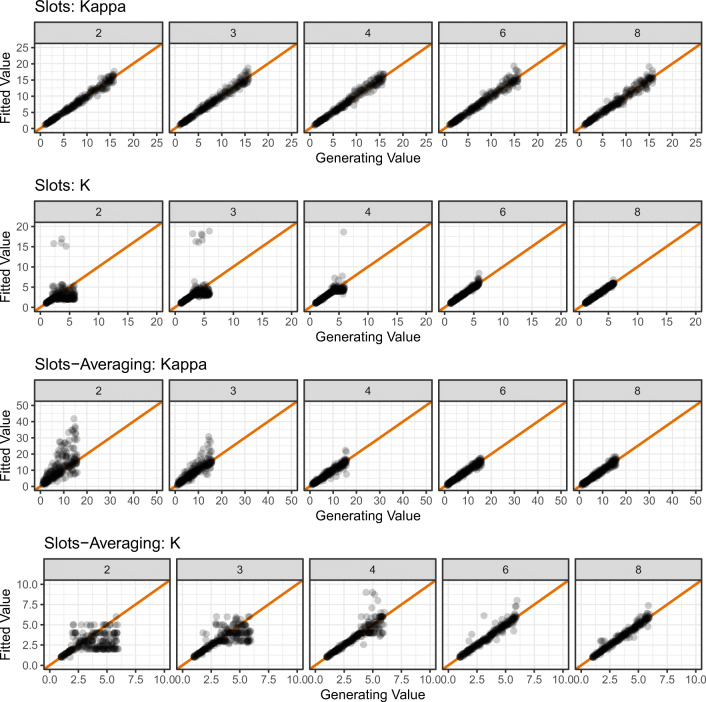
Results of parameter recovery simulations for the parameters of the three-component model as a function of the set size of stimulus display (i.e., number of memoranda). *Points* represent the best-fitting parameter values (*y*-axis) plotted against generating parameter values (*x*-axis) for 500 simulated participants. Column titles show the set size (i.e., number of memoranda) used in each simulation. The *diagonal line* represents perfect correspondence between generated and fitted parameter values

## Appendix B: Simulation Statistics

### B.1 Simulation 1: Parameter recovery

**Table 5:** Product–moment correlation coefficients for the parameter recovery of the two-component and three-component model simulation as a function of the number of trials

Model & Parameter	20	50	100	200	500	1000
Two-component (kappa)	.321	.822	.896	.960	.983	.992
Two-component (p_u)	.608	.844	.877	.871	.946	.975
Three-component (kappa)	-.036	.488	.796	.928	.972	.984
Three-component (p_u)	.449	.566	.679	.862	.899	.916
Three-component (p_n)	.576	.660	.779	.879	.938	.961

### B.2 Simulation 4: Set size

**Table 6:** Product–moment correlation coefficients for the parameter recovery of the two-component and three-component model simulation as a function of set size

Model & Parameter	2	3	4	6	8
Three-component (kappa)	.975	.976	.962	.963	.953
Three-component (p_u)	.933	.900	.883	.817	.850
Three-component (p_n)	.972	.957	.938	.866	.842

### B.3 Simulation 5: Memoranda similarity

**Table 7:** Product–moment correlation coefficients for the parameter recovery of the three-component model simulation as a function of the angular distance between items

Model & Parameter	5	10	15	20	30	40
Three-component (kappa)	.975	.962	.965	.966	.959	.962
Three-component (p_u)	.966	.938	.946	.947	.946	.914
Three-component (p_n)	.673	.883	.906	.923	.964	.964

## Appendix C: Slot Models

In this section, we describe the slots and slots-plus-averaging models as well as how to fit and simulate these models in mixtur. We then conduct various design simulations mirroring those in the main body of the article.

### C.1 Overview of models

#### Slots model

As described in the main body of the paper, the parameters for both the slots and slots-plus-averaging models are the number of slots (given by parameter *K*) and the precision of memory representations (given by parameter *κ*). If the current set size (given by *N*) is lower than or equal to capacity *K*, then all items are remembered and the participant’s response ($\hat \theta $) is modelled as a von Mises distribution centered on the true target value (*θ*),

7 $$ p(\hat\theta) = \phi_{\kappa}(\hat\theta - \theta). $$

However, when the set size *N* exceeds capacity *K*, the probed target will not always have been memorised by the participant. In such a case, the probed target is encoded with probability $\frac {K}{N}$; if a non-encoded item is probed then the participant will resort to guessing (which therefore occurs with probability $1 - \frac {K}{N}$). The participant’s response is therefore a mixture distribution of noisy responses to the true target value and guessing,

8 $$ p(\hat\theta) = \frac{\mathrm{K}}{\mathrm{N}}\phi_{\kappa}(\hat\theta - \theta) + \left( 1 - \frac{\mathrm{K}}{\mathrm{N}} \right) \frac{1}{2\pi}, $$

#### Slots-plus-averaging model

In the slots-plus-averaging model, items can be stored in multiple slots if capacity allows. At test, items stored in multiple slots are reported by using average of the stored representations (which reduces noise and therefore increases the accuracy of the response). When *N* is greater than capacity *K*, no item is stored in more than one slot, and responses are therefore a mixture of noisy responses to the true target value and guessing (as with the slots model),

9 $$ p(\hat\theta) = \frac{\mathrm{K}}{\mathrm{N}}\phi_{\kappa 1}(\hat\theta - \theta) + \left( 1 - \frac{\mathrm{K}}{\mathrm{N}} \right) \frac{1}{2\pi}, $$

where *κ*_1_ is the precision of items stored in a single slot. When capacity *K* is greater than *N*, at least one of the items will be stored in more than one slot (with probability $\frac {K mod N}{N}$. These items are therefore stored with higher precision (which we denote here as *κ*_*h**i**g**h*_) than items stored in a single slot (which we denote here as *κ*_*l**o**w*_). Responses are therefore a mixture distribution given by

10 $$ \begin{array}{@{}rcl@{}} p(\hat\theta) &=& \frac{\text{K mod N}}{\mathrm{N}}\phi_{\kappa high}(\hat\theta - \theta)\\ &&+ \left( 1 - \frac{\text{K mod N}}{\mathrm{N}} \right) \phi_{\kappa low}(\hat\theta - \theta). \end{array} $$

### C.2 Fitting & simulating the models

Although not essential for the model fitting procedure, both the slots model and the slots-plus-averaging model are best formed when the data has multiple set sizes. Unlike the components models, the slots models are fit to multiple set sizes at once with a single set of parameters.

To fit the models to data, the same fit_mixtur() function is used, placing either "slots" or "slots_averaging" in the model argument,

## id K kappa## 1 1 1.960 12.124## 2 2 1.788 9.577## 3 3 3.840 7.066## 4 4 3.535 11.017## 5 5 1.640 9.163

## 6 6 1.969 17.308

Likewise, to simulate data from either of the slots models, again the simulate_mixtur() function is used, placing either "slots" or "slots_averaging" in the model argument, but now the arguments K and kappa must be used. In addition, a vector of set sizes to use should be passed in the set_size argument.

The quality of the fit to data and the mean parameter values can be visualised using the plot_model_fit() and plot_model_parameters() function, respectively, again passing the correct model to the model argument.

### C.3 Design simulations

In this section, we repeat the simulations reported in the main body of the paper, but using the slots and slots-plus-averaging models.

#### Parameter recovery

This simulation repeats Simulation 1 from the main paper. We again simulated 500 synthetic participants, where the number of trials, *N*, used to simulate each participant varied across simulations (with the levels 20, 50, 100, 200, 500, and 1000). The parameters used to generate each participant’s data were randomly generated from a uniform distribution for each model parameter: *κ* (kappa) from 1 to 16 and *K* from 1 to 6. The results of theparameter recovery are shown in Fig. 13, and the correlation coefficients of the recovery are in Table 8.

**Table 8:** Product–moment correlation coefficients for the parameter recovery simulation as a function of the number of trials

Model & Parameter	20	50	100	200	500	1000
Slots (kappa)	.158	.466	.916	.954	.976	.991
Slots (K)	.723	.896	.900	.949	.984	.994
Slots_Averaging (kappa)	.280	.448	.724	.930	.971	.982
Slots_Averaging (K)	.764	.829	.898	.938	.960	.987

As can be seen, parameter recovery of *K* was very poor for both the slots and the slots-plus-averaging model below 100 trials, whilst kappa was recovered well with as few as 50 trials. Both *K* and kappa were recovered to an excellent level with just 100 trials for the slots model, 200 trials were required for both parameters to be recovered to an excellent level for the slots-plus-averaging model.

#### Parameter trade-off

This simulation mirrored Simulation 2 in the main paper. We systematically varied a single parameter across 50 levels whilst keeping the other parameter at a fixed level, and observed whether the parameter recovery shows a selective change in the varied parameter. Five hundred simulations were used for each level of parameter variation, with 500 trials being generated per simulation. For both the slots and the slots-plus-averaging models, the default model parameters were kappa = 8 and *K* = 3. kappa was varied between 4 and 12, and *K* was varied between 1 and 5. The results of the simulation are shown in Fig. 14. As can be seen in this figure, there was no evidence of a trade-off in model parameters between the varied parameter and the constant parameter. Thus the fit routine correctly identifies selective changes to individual parameters.

#### Model recovery

This simulation mirrored Simulation 3 in the main paper. We simulated data from a “true” model (either the slots or the slots-plus-averaging model, in separate simulations), and then fit both models to the same data set to see whether the true generating model fits the data better than the other model. For each model, we simulated 1000 data sets with 500 trials in each data set. The parameters used to generate each data set were randomly selected from a uniform distribution (kappa was between 1 and 16, and *K* was between 2 and 6; note that when *K* equals 1, the slots-plus-averaging model is identical to the slots model, so this value was not allowed). The frequency distributions of the difference in AIC and BIC scores are shown in Fig. 15.

As can be seen, when the slots model was the true generating model, both the AIC and BIC were able to correctly recover this model in the majority of simulations. The slots model was correctly recovered in 88.9% of the simulations via both AIC and BIC. When the slots-plus-averaging model was the true generating model, both AIC and BIC recovered the correct model 85.5% of the time. Thus, model recovery is generally very good.

#### Effect of set size

This simulation mirrored Simulation 4 in the main paper. As the slots and slots-plus-averaging model parameters account for performance across multiple set sizes, it is important to understand whether parameter estimation quality changes with the number of set sizes used in an experiment. We therefore explored parameter recovery quality across set sizes of 2, 3, 4, 6, and 8. Unlike the simulations reported in the main paper, set size here refers to the maximum set size in the simulated data, and data were therefore generated using set sizes from 1:N. For example, for a set size of 4, data were simulated from an experiment presenting set sizes of 1, 2, 3, and 4.

For each set size, we simulated 500 trials with model parameters randomly generated. We then fit the generating model back to the simulated data, and compared the recovered parameter values with the generating parameter values. This process was the repeated 500 times.

The results of the simulation are shown in Fig. 16 and the product–moment correlation coefficients are shown in Table 9. As can be seen, for both models recovery of kappa was generally good/excellent with a set size of just 2. However, *K* required more set size variations in order to be recovered well. For both models,

**Table 9:** Product–moment correlation coefficients for the parameter recovery simulation of the slots and the slots-plus-averaging models as a function of set size

Model & Parameter	2	3	4	6	8
Slots (kappa)	.990	.988	.989	.985	.985
Slots (K)	.184	.379	.769	.980	.991
Slots_Averaging (kappa)	.714	.870	.958	.979	.979
Slots_Averaging (K)	.443	.747	.895	.969	.980

a set size of 4 was required to recover *K* to a good level, and 6 was needed for an excellent level. (We did not explore a set size of 5, so this may well have produced excellent recovery.) The results of this simulation suggest that multiple set sizes should be used in an experiment wishing to fit the slots and the slots-plus-averaging model to estimate capacity *K* accurately. This is not a surprising outcome; capacity cannot be established if just one value for set size is used.

### C.4 Slots-plus-resources model

The slots-plus-resources model (Zhang & Luck, 2008) assumes that vSTM contains a fixed number of slots. The main difference with this model compared to the slots and slots-plus-averaging models is that a memory resource can be variably allocated to each slot. The amount of resource allocated to each slot therefore determines the precision with which representations within each slot are retained in memory: A slot receiving minimal resource will contain a low precision representation, whereas a slot receiving substantial resource will contain a high precision representation. However, Zhang and Luck (2008) provided evidence to rule out the existence of such a model. By presenting a mask 110 ms after stimulus presentation in a delayed estimation task, Zhang and Luck showed that the probability that an item was represented in memory was reduced, but the precision of representations that were stored in memory remained stable. The authors state that this provides evidence in support of the view that an “all-or-none” step is required to generate robust internal representations of a fixed resolution. This model is therefore not implemented in mixtur.

## Footnotes

1 It is important to note that the assumption of equal distribution of resources across all items in the display as stated by Bays et al., (2009) is not true of all mixture models, such as the variable precision model of van den Berg, Shin, Chou, George, and Ma (2012).

2 Note that mixtur does not do the analysis for the user. We decided against including this as a feature in the package so that the software remains agnostic as to the best way to analyse data (i.e., to remain neutral in the Bayes vs. frequentist debate).

3 These are available at https://www.paulbays.com/toolbox/index.php and https://www.cns.nyu.edu/malab/resources.html, respectively.

4 We, furthermore, used the R-packages *dplyr* (Version 1.0.6, Wickham et al., 2021), *forcats* (Version 0.5.0 , Wickham, 2020), *ggplot2* (Version 3.3.3, Wickham, 2016), *gridExtra* (Version 2.3, Auguie, 2017), *here* (Version 1.0.1, Müller, 2017), *kableExtra* (Version 1.2.1, Zhu, 2020), *papaja* (Version 0.1.0.9997, spiciteAust-and-Barth:2020), *purrr* (Version 0.3.4, Henry & Wickham, 2020), *RColorBrewer* (Version 1.1.2, Neuwirth, 2014), *readr* (Version 1.3.1, Wickham, Hester, & Francois, 2018), *stringr* (Version 1.4.0, Wickham, 2019), *tibble* (Version 3.1.1, Müller & Wickham, 2021), *tidyr* (Version 1.1.3, Wickham, 2021), *tidyverse* (Version 1.3.0, Wickham et al., 2019), and *tinylabels* (Version 0.1.0, Barth, 2020).

5 The data from Bays et al., (2009) are publicly available at https://osf.io/c2yx5/, and the Berry et al., (2019) data are available at https://osf.io/59c4g/.

6 See Appendix A for a sample script that can be used if the user wishes to plot the model fit per participant to see if any individual participant’s data was not well fit by the model.

7 Note that we explored other default parameter values to make sure our findings were robust to our choice of starting values. These additional analyses were qualitatively similar to that reported here.
